# Computational analysis of mixed cation mixed halide-based perovskite solar cell using SCAPS-1D software

**DOI:** 10.1016/j.heliyon.2022.e11428

**Published:** 2022-11-07

**Authors:** A.M. Ntouga Abena, A. Teyou Ngoupo, J.M.B. Ndjaka

**Affiliations:** Université de Yaoundé 1, Faculté des Sciences, Département de Physique, BP 812, Yaoundé, Cameroon

**Keywords:** Hybrid perovskite, Strontium, Formamidinium, Substitution, Numerical simulation, SCAPS-1D

## Abstract

Standard MAPbI_3_ (MAPI) perovskite suffers from stability and toxicity problems. In this numerical simulation study using SCAPS-1D software, we propose a hybrid perovskite (MA_1−*x*_FA_*x*_Pb_1−*y*_Sr_*y*_I_3_) to reduce these effects; thus, the influence of the mixture of formamidinium (NH_2_CHNH${}_{2}^{+}$ (FA^+^)), strontium (Sr), methylammonium (CH_3_NH${}_{2}^{+}$ (MA^+^)) and lead (Pb) on the electrical parameters of a hybrid perovskite-based solar cell is studied. This simulation was performed through modeling the perovskite absorber band gap depending on *x* and *y* proportions. This mixture leads to increase the crystallinity or stability by decreasing MA^+^ proportion by FA^+^, while the toxicity is reduced by decreasing Pb^2+^ proportion by Sr^2+^. We show that the substitution of 90% MA and 15% Pb (MA_0.1_FA_0.9_Pb_0.85_Sr_0.15_I_3_) to the standard MAPI radically changes the electrical parameters of the material and the performance of the solar cell. A maximum efficiency of 29% ($J_{sc}=24.2$ mA/cm^2^, $V_{oc}=1.37$ V, FF=87.49%) is obtained in this simulation of the hybrid perovskite-based solar cell. These results are obtained after optimizing the hybrid perovskite band gap (Eg = 1.60 eV), layer thicknesses (0.400 μm for hybrid perovskite, 0.250 μm for TiO_2_ ETL, and 0.150 μm for Cu_2_O HTL), absorber bulk defect density (10^13^ cm^−3^), and perovskite/TiO_2_ interface defects density (10^12^ cm^−2^). Our results show that the composition of MA, FA, Pb, and Sr in the MA_1−*x*_FA_*x*_Pb_1−*y*_Sr_y_I_3_ hybrid perovskite may be a way to obtain new perovskites with interesting physical properties for application in solar cells.

## 1 Introduction

Organic-inorganic perovskites have received considerable attention in recent years. This is due to their great potential as new optoelectronic materials for devices that could be processed with simple and cheap techniques on large surfaces and flexible substrates (Docampo et al., 2013; Hu et al., 2014) and to the modulation of their intrinsic properties (i.e. by changing the composition of AMX_3_ lattice, where A^+^ is an organic and/or inorganic cations (Cs^+^, methylammonium (CH3NH=3+MA+), formamidinium (HC(NH2)=2+FA+)), M^2+^ is a metal cation, and X^−^ is a halide anion (Cl^−^, Br^−^, I^−^)). The discovery of their optoelectronic properties has led to the synthesis of many lead halide perovskite materials (APbX_3_). A large number of mixed perovskites, i.e., with a mixture of homovalente organic and/or inorganic cations (McMeekin et al., 2016; Pellet et al., 2014; Saliba et al., 2016) and/or a mixture of homovalents anions, have also been successfully synthesized. These perovskite materials have been developed to improve the performance and stability of photovoltaic devices using these types of materials as an absorber layer. However, lead toxicity remains a major issue for the further development of halogenated perovskite-based photovoltaic devices (Yin et al., 2014). Indeed, the degradation of materials in the presence of humidity leads to the release of ${\mathrm{Pb}}^{2+}$ in aqueous medium, posing serious health and environmental problems. Thus; many studies have focused on the substitution of homovalent Pb^2+^ cation by Sn^2+^ cation (Eperon et al., 2016; Noel et al., 2014), since it belongs to the same group in the periodic table and therefore materials with similar properties are expected; but, Sn^2+^ easily oxidizes to Sn^4+^ when exposed to air (Adjokatse et al., 2019), which compromises the stability of perovskite materials and renders them unusable. Therefore, the identification of alternative divalent metal cations to Pb^2+^ and Sn^2+^ and allowing to preserve the excellent optoelectronic properties of perovskite, to reduce its toxicity without further altering its stability, remains to be explored. Thus, compositional engineering and doping are commonly employed strategies, which are viable means to control the crystal growth, structural stability, and light conversion properties of most perovskite materials (Lau et al., 2017; Zhou et al., 2018). Strontium (Sr^2+^) is one of the metal dopants with multiple functions such as improving the stability of the host perovskite and the device performance; in addition, its oxidation state (+2) allows the doping the crystal structure of Sn-based perovskites to stabilize and tune their optoelectronic properties. Moreover, strontium is very abundant and environmentally friendly (Adjokatse et al., 2019). Its ionic radius (1.18 Å) is close to that of Pb^2+^ (1.19 Å) (Kour et al., 2019), which favors an excellent substitution of lead while maintaining the intrinsic properties of the host (Gualdrón-Reyes et al., 2021). However, pure Sr halide perovskite has a very wide band gap (3.6 eV for MASrI_3_ (Pérez-del-Rey et al., 2016)) which is not suitable for the absorber layer in single n-p junction solar cells, as their band gap limit is 1.60 eV (Polman et al., 2016).

It is also necessary to remedy the crystallinity problem caused using the organic cation MA^+^ in the perovskite and in this case, formamidinium (HC(NH2)=2+FA+) is used to promote a more stable perovskite structure (Mateen et al., 2020). However, the implementation of the device with FAPbI_3_ as absorber being very restrictive (Mozaffari et al., 2018), and the resulting performances are often lower than those of the MAPbI_3_-based solar cell. To solve these problems, composition engineering suggests the mixing of MA^+^ and FA^+^ cations as in the structure proposed by He et al. (MA_1−*x*_FA_*x*_PbI_3_) (He et al., 2017). In this simulation study via SCAP-1D software, we employ compositional engineering through the hybrid perovskite structure MA_1−*x*_FA_*x*_Pb_1−*y*_Sr_y_I_3_ to propose a solution to both crystallinity and toxicity problems; while optimizing the electrical parameters of the cell. First, we determine the correlation between the band gap and the different proportions *x* and *y*. Then, we solve the equation obtained by varying these different ratios to obtain different values of the band gap. Finally, we run simulations with these different band gaps to see their impact on the performance of the device. In practice, the implementation of the partial *x* and *y* substitutions of the cations can be done by a combination of different techniques such as the two-step spin coating used by He et al. (2017), doping engineering used by Gualdrón-Reyes et al. (2021) and Adjokatse et al. (2019) for *y* proportions. Furthermore, the formation of AMX_3_ halide perovskites depends on:

(i) The stability of the BX_6_ octahedra, which can be predicted by the octahedral factor *μ*,
(ii) The ionic radii of A, B, and X must satisfy the Goldschmidt tolerance factor *t*.

According to Hoefler et al. (2017), Travis et al. (2016), Jacobsson et al. (2015), both of these criteria are satisfied by the perovskite absorber used in our device. Based on the band gap limit and the available experimental data on strontium (Sr), we show that the suitable absorber perovskite layer is MA_0.1_FA_0.9_Pb_0.85_Sr_0.15_I_3_, with a band gap of 1.60 eV. The optimal electrical parameters of the solar cell based on this perovskite absorber layer are: Jsc = 24.2 mA/cm^2^, V_oc_ = 1.37 V, FF = 87.49%, and PCE = 29%.

## 2 Modeling and structure of the solar cell

### 2.1 Numerical modeling

The concept of the numerical simulation of a solar cell is based on the resolution of the differential equations of Poisson and continuity in semiconductors (Abena et al., 2022; Ngoupo et al., 2021). These equations are nonlinearly coupled differential equations for both electrons and holes and are position dependent. In view of the size of the cells, of the order of a few micrometers for the largest, a one-dimensional numerical resolution of these equations is realistic enough to produce convincing results. In this work, we have adopted a one-dimensional description of the perovskite-based solar cell, using the simulation code SCAPS-1D, developed by Burgelman et al. (2000).

### 2.2 SCAPS input parameters

**Figure 1:**
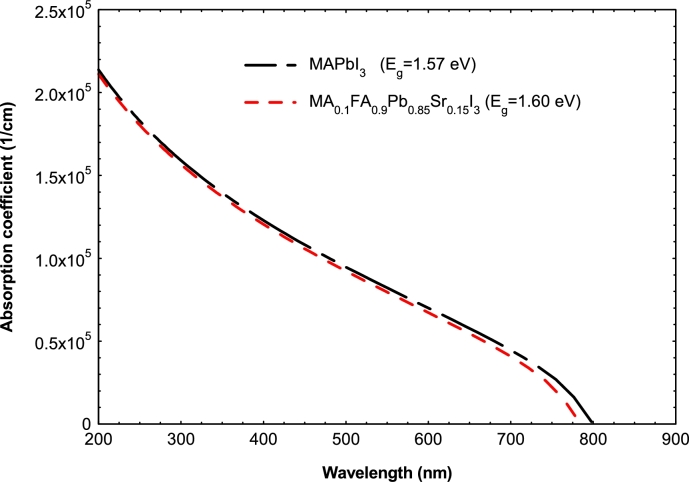
Absorption coefficient of different configurations of the absorber layer.

The structure of our basic solar cell is Glass/SnO_2_:F/TiO_2_/MA_0.5_FA_0.5_PbI_3_/Cu_2_O/Au, as we presented and explained in our previous work (Abena et al., 2022). However, to propose a solution to the crystallinity and toxicity problems of perovskite, the absorber layer MA_0.5_FA_0.5_PbI_3_ is replaced by MA_1−*x*_FA_x_Pb_1−*y*_Sr_y_I_3_. The optical absorption coefficient of the absorber is calculated using the following relationship $\alpha \left(E\right)=A_{\alpha }{\left(h\nu -E_{g}\right)}^{0.5}$ (Gamal et al., 2021), where the pre-factor $A_{\alpha }$ is 10^5^ cm^−1^eV^−0.5^. This absorption coefficient is thus affected by the band gap which varies with *x* and *y*. When the band gap increases, the absorption coefficient decreases and this is illustrated in Fig. 1. The input parameters of the solar cell are presented in Table 1.

**Table 1:** Input parameters in SCAPS-1D.

Layers	TCO	ETL	Perovskite	HTL
	SnO_2_:F (FTO)	TiO_2_	MA_0.5_FA_0.5_PbI_3_	Cu_2_O
Thickness (nm)	500 (Tan et al., 2016)	50 (Variable)	600 (Variable)	150 (Abena et al., 2022)
Dielectric constant (relative)	9 (Tan et al., 2016)	9 (Ngoupo et al., 2021)	6.5 (Hirasawa et al., 1994; Slami et al., 2019)	7.11 (Hossain et al., 2015)
Electron mobility (cm^2^/V.s)	20 (Tan et al., 2016)	20 (Ngoupo et al., 2021)	2 (Tan et al., 2016)	200 (Sawicka-Chudy et al., 2017)
Hole mobility (cm^2^/V.s)	10 (Tan et al., 2016)	10 (Ngoupo et al., 2021)	2 (Tan et al., 2016)	80 (Sawicka-Chudy et al., 2017)
Acceptor concentration (1/cm^3^)	0 (Tan et al., 2016)	0 (Tan et al., 2016)	10^9^ (Si et al., 2016)	6×10^18^ (Abena et al., 2022)
Donor concentration (1/cm^3^)	10^15^ (Abena et al., 2022)	6×10^18^ (Abena et al., 2022)	10^9^ (Si et al., 2016)	0 (Abena et al., 2022)
Band gap (eV)	3.5 (Tan et al., 2016)	3.3 (Ngoupo et al., 2021)	1.48 (Abena et al., 2022)	2.17 (Sawicka-Chudy et al., 2017)
*N*_*C*_ (1/cm^3^)	2.2 × 10^17^ (Abena et al., 2022)	2 × 10^17^ (Sawicka-Chudy et al., 2017)	2.2×10^15^ (Abena et al., 2022)	2.02 × 10^17^
*N*_*V*_ (1/cm^3^)	2.2 × 10^16^ (Abena et al., 2022)	1 × 10^17^	2.2×10^17^ (Abena et al., 2022)	1.1 × 10^19^ (L. Zhu et al., 2011)
Electron affinity (eV)	4 (Tan et al., 2016)	3.9 (L. Zhu et al., 2011)	3.758 (Abena et al., 2022)	3.20 (L. Zhu et al., 2011)

## 3 Results and discussion

Firstly, we correlate the *x* and *y* proportions with the band gap Eg. Thus, by solving the equation Eg(*x*, *y*) obtained, we simultaneously obtain the rates of MA and Pb which can be substituted, and the corresponding band gap value. Subsequently, we perform simulations of this Glass/SnO_2_:F/TiO_2_/MA_0.1_FA_0.9_Pb_0.85_Sr_0.15_I_3_/Cu_2_O/Au solar cell using the SCAPS-1D software to determine the optimal photovoltaic parameters.

### 3.1 Effect of formamidinium (FA) and strontium (Sr) proportions on the perovskite solar cell performance

Shockley and Queisser showed that the band gap (Eg) of the absorber material used in solar cells strongly influences the photovoltaic performance (Shockley and Queisser, 1961). However, Eg often varies nonlinearly with the composition (*x*) of ternary alloys ($A_{x}B_{1-x}C$) (J. Xu et al., 2011), and as a first approximation, its variation is quadratic (Equation (1)) (Swafford et al., 2006; Venugopal et al., 2006; Wang et al., 2007).

(1) $$E_{g}\left(A_{x}B_{1-x}C\right)=xE_{g}\left(AC\right)+(1-x)E_{g}\left(BC\right)-bx(1-x)$$

where *b* is a curvature parameter, describing the extent of the nonlinearity.

In this numerical study, by applying the band gap variation law (Equation (1)) to the work of Ono et al. (2017), Gualdrón-Reyes et al. (2021) and Navas et al. (2015), we respectively obtain the variation of the band gaps of the perovskite absorbers MA_1-x_FA_x_PbI_3_ (Equation (2)), FAPb_1−*y*_Sr_y_I_3_ (Equation (3)) MAPb_1−*y*_Sr_y_I_3_ (Equation (4)), with a (*x*) FA content and (y) Sr content.

(2) $$E_{g}(eV)=0.074x^{2}-0.155x+1.54$$

with x∈[0;1].

(3) $$Eg(eV)=1.98y^{2}+0.43y+1.59$$

with y∈[0;0.12].

(4) $$Eg(eV)=7y^{2}-1.43y+1.57$$

with y∈[0;0.15].

Thus, the application of Equations (1) to (4) to the hybrid perovskite MA_1−*x*_FA*x*Pb_1-y_Sr_y_I_3_ leads to the following band gap variation law:

(5) $$Eg(eV)=0.074x^{2}+7y^{2}-5.02xy^{2}+1.86xy-0.05x-1.43y+1.57$$

with x∈[0;1] and y∈[0;0.15]

**Figure 2:**
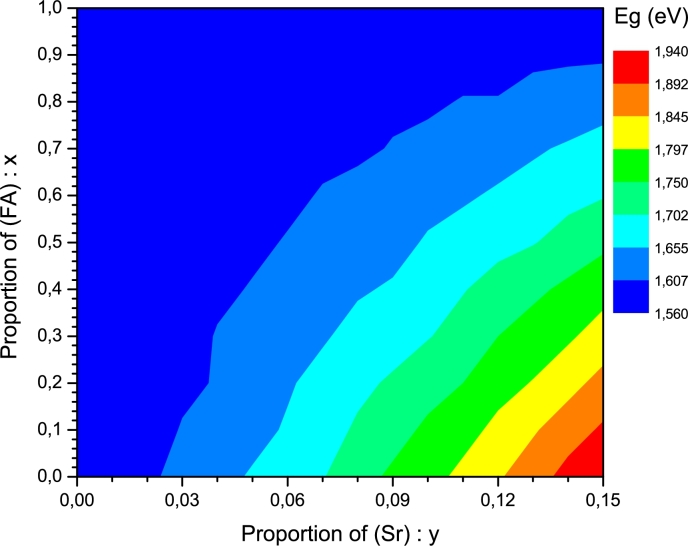
Effect of the proportions (*x*) and (*y*) on the band gap of the MA_1−*x*_FA_*x*_Pb_1-y_Sr_y_I_3_ absorber.

**Figure 3:**
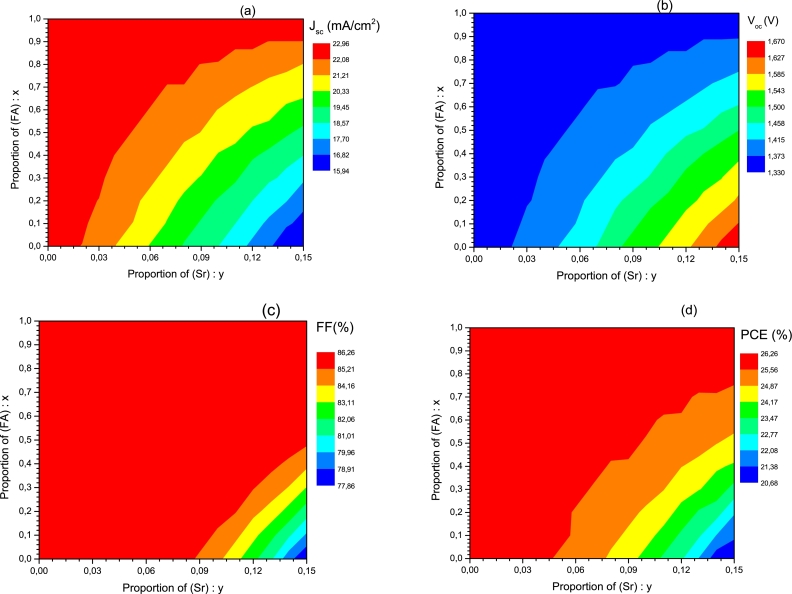
Variation of electrical parameters as a function of proportions (x) and (y). (a) *J*_*sc*_, (b) *V*_*oc*_, (c) *FF*, and (d) efficiency (*PCE*).

Fig. 2 shows, according to Equation (5), the influence of the proportions of FA and Sr on the hybrid perovskite band gap. It can be observed that the band gap increases when the strontium concentration (*y*) increases and the formamidinium concentration (*x*) decreases. The ideal is to decrease the band gap, it is thus beneficial to substitute MA by FA because as *x* increases, the band gap decreases. On the other hand, the band gap increases as *y* increases, despite the fact that it is necessary to substitute Pb by Sr. It is probably through this joint substitution that the negative effect of the partial replacement of lead is reduced. This shows that the smaller the band gap, the higher some electrical parameters. However, for an FA content greater than or equal to 0.9 (90%), and whatever the proportion of Sr, the band gap is less than or equal to 1.60 eV (Fig. 2), which corresponds to the Shockley-Queisser limit, in the case of the perovskite absorber for n-p single junction solar cells (Polman et al., 2016). For an adequate substitution of lead (Pb) by strontium (Sr) and in agreement with the above-mentioned band gap limit, 15% of Sr is the optimal proportion, which allows the perovskite absorber MA_0.1_FA_0.9_Pb_0.85_Sr_0.15_I_3_ to be obtained. This perovskite structure could be obtained by improving the quality and method of deposition. The choice of the band gap value at 1.60 eV, corresponding to 90% FA and 15% Sr, is confirmed by Fig. 3, which illustrates the dependence of the electrical parameters on the proportions *x* and *y*.

**Figure 4:**
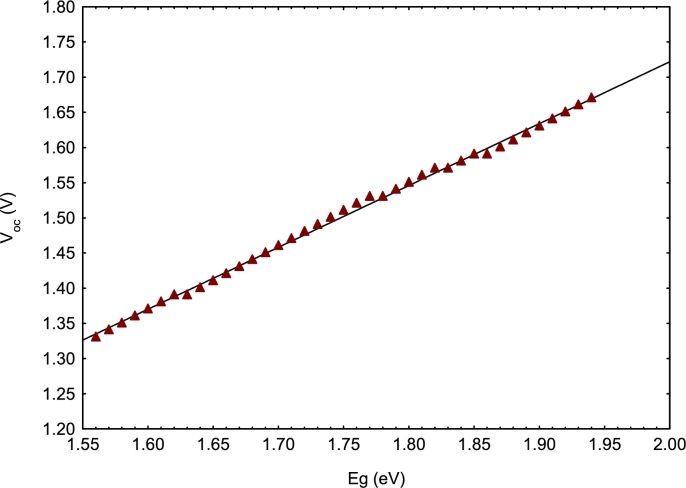
Variation of *V*_*oc*_ as a function of the band gap of the MA_1-*x*_FA_*x*_Pb_1-y_Sr_y_I_3_ absorber.

Fig. 3 shows that for a proportion *x* greater than or equal to 90%, and whatever the concentration *y*, the electrical parameters $J_{sc}$ (Fig. 3.a), *FF* (Fig. 3.c), and *PCE* (Fig. 3.d) are optimal. Thus, we can reasonably choose y=0.15 which is the highest value proportion of Sr in this study. This corresponds to a band gap less than or equal to 1.60 eV as mentioned previously. On the other hand, only *V*_oc_ presents a variation contrary to the other electrical parameters according to the proportions *x* and *y* (Fig. 3.b), and similar to that of Eg (Fig. 2). This suggests a correlation between the band gap and the open circuit voltage; it is a linear correlation justified by Fig. 4 and Equation (6). This equation is obtained by linear fitting. Many studies have also shown that there is a linear correlation between Voc and band gap, among these are Yang et al. (2017), Vandewal et al. (2008), and Scharber et al. (2006).

(6) $$V_{OC}=0.879\times E_{g}-0.0368$$

### 3.2 Effect of perovskite and ETL layer thicknesses

**Figure 5:**
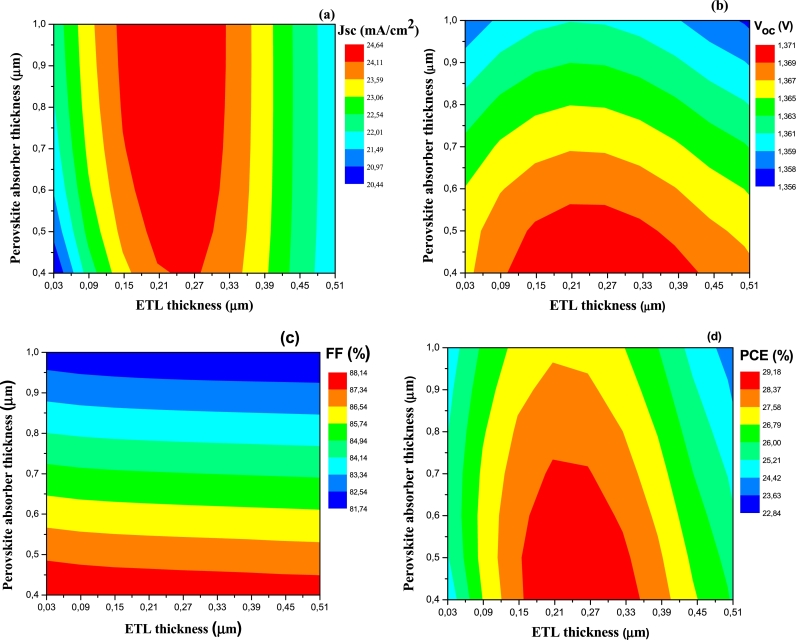
Variation of electrical parameters as a function of absorber and ETL thicknesses. (a) *J*_*sc*_, (b) *V*_*oc*_, (c) *FF*, and (d) efficiency (*PCE*).

An absorber layer is crucial in improving device performance (Ngoupo et al., 2021; Rai et al., 2020). All absorber parameters, such as thickness (Abena et al., 2022), band gap, doping concentration, and defects, are important in optimizing device performance (L. Lin et al., 2019). In the same way, the performance of solar cells is sensitive to the thickness of the electron transport layer (ETL) (Jeyakumar et al., 2020). To examine the influence of these thicknesses, the simulation is performed for different thicknesses ranging from 0.4 μm to 1 μm for the absorber, and from 0.03 μm to 0.51 μm for ETL, keeping constant all other parameters of Table 1. We observe that the $J_{sc}$ (Fig. 5.a), $V_{oc}$ (Fig. 5.b), and *PCE* (Fig. 5.d) of the device, first increase when the ETL thickness increases up to limit values; this could be due to the fact that increasing the ETL thickness leads to a reduction in the leakage current ($J_{\mathrm{surf}}$) at the perovskite/ETL interface (Equation (7)) (An et al., 2018).

(7) $$J_{sc}=J_{ph}-J_{bulk}-J_{surf}$$

Where $J_{ph}$ is the photo generated current density, $J_{sc}$ is the short circuit current density, $J_{\mathrm{bulk}}$ is the current loss due to bulk recombination, and $J_{\mathrm{surf}}$ is the current loss due to surface recombination.

On the other hand, beyond each of the ETL thickness limit values, these three electrical parameters ($J_{sc}$ (Fig. 5.a), $V_{oc}$ (Fig. 5.b), and *PCE* (Fig. 5.d)) decrease, because a greater number of photons are absorbed in this layer and do not contribute to the generation of charge carriers, hence the decrease of $J_{sc}$; moreover, this increase in thickness induces that of the recombination current $J_{0}$. The combination of these two phenomena leads to a decrease of $V_{oc}$ (Equation (8) Rai et al., 2020). The decrease of *PCE* is due to the joint decrease in $J_{sc}$ and $V_{oc}$ according to Equation (9). *FF* decreases with increasing ETL thickness (Fig. 5.c) due to the increase of charge carrier recombination.

(8) $$V_{oc}=\frac{nKT}{q}\ln\left[\frac{J_{sc}}{J_{0}}+1\right]$$

Where $\frac{KT}{q}$ is the thermal voltage and *n* is a factor due to the increase in series resistance.

(9) $$PCE=V_{oc}\times J_{sc}\times FF/P_{in}$$

Furthermore, we observe a slight increase of $J_{sc}$ with the increase of the thickness of the absorber (Fig. 5.a). This result is explained by a slight increase in the thickness of the space charge region (*W*) with the absorber thickness, which increases the photocurrent through Equation (10) (Abega et al., 2021); and thus causes $J_{sc}$ to increase through Equation (7).

(10) $$J_{ph}=q\times G\times \left(L_{n}+W+L_{p}\right)$$

Where $L_{n}$ and $L_{p}$ are the electron and hole diffusion lengths, respectively, *W* is the width of the space charge region, *q* is the elementary charge, and *G* is the carrier generation rate.

The decrease of $V_{oc}$ with increasing absorber thickness (Fig. 5.b), is a result of the increase of the recombination phenomenon (Cho and Park, 2017; Jeyakumar et al., 2020) represented by $J_{0}$ in Equation (8). Fig. 5.c shows that increasing perovskite layer thickness induces a decline of FF from 88.14% to 81.74%, this is explained by the relation between *FF* and $V_{oc}$ (equation (11) (Guirdjebaye et al., 2019); thus, the increase in the recombination rate and series resistance with this thickness induces a decrease in the *FF*.

(11) $$FF=\frac{v_{oc}-\ln\left(v_{oc}+0.72\right)}{v_{oc}+1}$$

Where $v_{oc}=\frac{qV_{oc}}{AK_{B}T}$

Moreover, as the absorber thickness increases, the charge carriers have also to cover a greater distance to reach the electrodes, increasing the probability of electron recombination with the minority carriers (holes); on the other hand, the low mobility of electrons in the TiO_2_ layer accentuates this effect. Consequently, the efficiency of the cell decreases (Fig. 5.d) and is accentuated by the decrease of $V_{oc}$ and *FF*. The maximum efficiency of 29.18% is obtained for absorber and ETL thicknesses equal to 0.400 μm and 0.250 μm, respectively. We therefore choose these thickness values for the rest of our work.

### 3.3 Influence of absorber defect density

**Figure 6:**
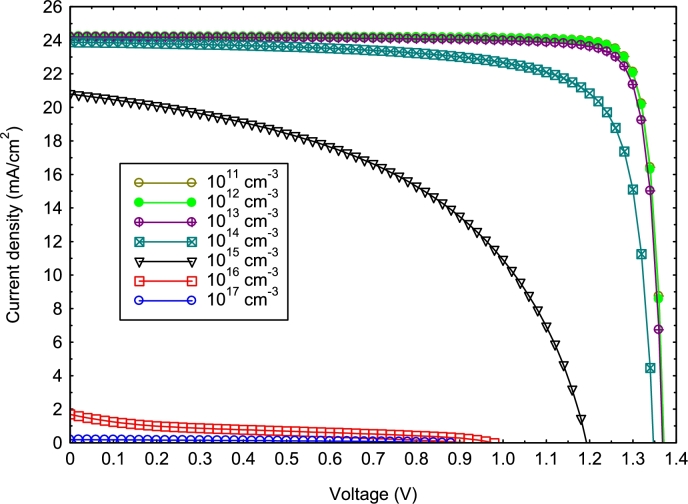
J-V characteristics of the perovskite solar cell as a function of absorber defect density.

To estimate the optimal defect concentration in the absorber for optimal electrical parameters, a simulation was realized, varying the defect density from 10^11^ cm^−3^ to 10^17^ cm^−3^, as in the work of Shahariar et al. (2020). The J-V characteristic curve (Fig. 6) illustrates how the device parameters decrease significantly when the defect concentration in the absorber is greater than 10^13^ cm^−3^. Thus, to obtain a better efficiency, the defects in perovskite must be reduced to 10^13^ cm^−3^, as in the work of Slami et al. (2019). This could be done by improving the crystal structure and the processing method. A promising *FF* and *PCE* of 87.49% and 29.00% were obtained at a defect density of 10^13^ cm^−3^.

A higher defect density leads to a higher recombination rate in the perovskite layer, which reduces the carrier lifetime as well as the diffusion length ($L_{D}$) of the charge carriers (Jamal et al., 2019; Sridharan et al., 2019), as shown in Equations (12) and (13) (Green, 1982). This provides a theoretical explanation for the mechanisms that cause the reduction of the electrical parameters of the solar cell.

(12) $$\tau_{lifetime}=\frac{1}{N_{t}\delta v_{th}}$$

(13) $$L_{D}=\sqrt{\frac{\mu_{\left(e,h\right)}ℜT}{q}\tau_{lifetime}}$$

where, $N_{t}$, *δ*, $v_{th}$, $L_{D}$, $\mu_{\left(e,h\right)}$, ℜ, *T*, *q*, and $\tau_{lifetime}$ are, respectively the defect density, electron and hole capture cross-section, thermal velocity of the carrier, diffusion length, electron and hole mobility, Boltzmann constant, temperature, charge, and carrier lifetime.

Based on Equations (8) and (14), we can conclude that increasing defect density can increase the recombination current and lead to a decrease in $V_{oc}$. This is in agreement with the results of Chowdhury et al. (2020), MaríSoucase et al. (2016), and Jamal et al. (2019). Furthermore, the decrease in the internal quantum efficiency (*IQE*) is due to the increasing of the diffusion length ($L_{D}$), according to Equation (15) (Geist, 1979); this has a negative effect on the $J_{sc}$ (Fig. 6).

(14) $$J_{0}\approx q\frac{Dn_{i}^{2}}{L_{D}N_{t}}$$

(15) $$IQE=1-\alpha t-\frac{B}{\alpha L_{D}^{2}}$$

where *α*, *t*, and *B* are, respectively, the spectral absorption coefficient, the distance to the perovskite material, and the thickness of the perovskite.

### 3.4 Effect of perovskite/TiO_2_ interface defect density on PV parameters of PSC

**Figure 7:**
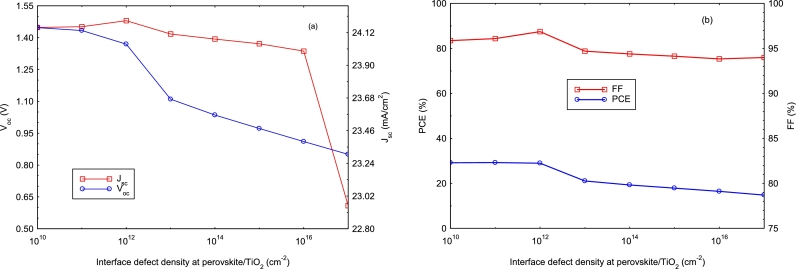
Variation of *J*_*sc*_, *V*_*oc*_, *FF*, and efficiency (*PCE*) as a function of perovskite/TiO_2_ interface defects. (a) *V*_*oc*_ and *J*_*sc*_, (b) *PCE* and *FF*.

The structural incompatibility of two different materials leads to the occurrence of interfacial defects. The quality of the junction is therefore essential for the performance of the photovoltaic device. In this simulation study, the defect densities at the perovskite/TiO_2_ interface vary from 10^10^ cm^−2^ to 10^17^ cm^−2^ and Fig. 7 shows the effects of this variation on the device performance. It can be seen that for a defect density of 10^10^ cm^−2^ to 10^12^ cm^−2^, the device parameters vary very little; but beyond 10^12^ cm^−2^, they start to decrease due to the fact that interface defects behave as recombination centers (Ahmed et al., 2021). Moreover, the largest generation of electron-hole pairs occurs at the perovskite/TiO_2_ interface; which is also accompanied by a higher recombination rate (Sengar et al., 2021). We choose an optimal defect density equal to 10^12^ cm^−2^ and this leads to the following electrical parameters: $J_{sc}=24.2$ mA/cm^2^, $V_{oc}=1.37$ V, FF=87.49%; and *PCE* = 29%.

Comparing our simulation results with the experimental ones found in the literature (Table 2), we can observe that our results are better than those of the experiment. This is due to the fact that numerical simulation allows to reach unexplored limits by experiment. Thus, numerical simulation is a promising strategy for investigating the properties of photovoltaic devices to improve their performance. The partial substitution of lead by strontium (up to 15%) is a strategy to improve the open-circuit voltage and the fill factor of the hybrid perovskite-based solar cell.

**Table 2:** Comparison of the performance of photovoltaic devices based on different perovskite absorbers.

Strategy	Perovskite absorber	*J*_*sc*_ (mA/cm^2^)	*V*_*oc*_ (V)	*FF* (%)	*PCE*
Simulation	MA_0.1_FA_0.9_Pb_0.85_Sr_0.15_I_3_	24.2	1.37	87.49	29
	(This work)				
Experimental	MA_0.3_FA_0.7_Pb_0.5_Sn_0.5_I_3_	31.4	0.83	81	21.1
	(R. Lin et al., 2019)				
	MA_0.6_FA_0.4_Sn_0.6_Pb_0.4_I_3_	30.5	0.83	81	20.5
	(Tong et al., 2019)				
	FA_0.5_MA_0.45_Cs_0.05_Pb_0.5_Sn_0.5_I_3_	30.2	0.85	79	20.3
	(Yang et al., 2019)				
	Cs_0.025_FA_0.475_MA_0.5_Sn_0.5_Pb_0.5_I_3_	33.14	0.81	76	20.40
	(Kapil et al., 2019)				
	MA_0.5_FA_0.5_Pb_0.5_Sn_0.5_I_3_	25.69	0.78	70	14.01
	(X. Xu et al., 2017)				
	FA_0.85_MA_0.15_Pb_0.6_Sn_0.4_(I_0.85_Br_0.15_)_3_	26.45	0.87	79	18.21
	(Z. Zhu et al., 2019)				
	FA_0.75_MA_0.25_Sn_0.95_Ge_0.05_I_3_	19.5	0.42	55	4.48
	(Ito et al., 2018)				

### 3.5 Effect of temperature on solar cell performance

Solar cells are largely governed by their operating temperature. To determine the temperature dependence of our device's performance, we performed the solar cell parameters using the SCAPS-1D solar simulator, at ambient temperatures (*T*) ranging from 25 °C to 105 °C with a 10 °C increment, under 1 sun irradiation as in our previous work (Abena et al., 2022). The value of the temperature coefficient (TC) can be expressed as follows (Krajangsang et al., 2014):

(16) TC(par/∘C)=1Z×(δZδT)|Tn=25C∘

where *Z* means solar cell parameters such as efficiency (*PCE*), open circuit voltage ($V_{oc}$), short circuit current ($J_{sc}$), and fill factor (*FF*). With a standard temperature ($T_{n}$) equal to 25 °C, corresponding to the standard measurement of the test solar cells.

**Figure 8:**
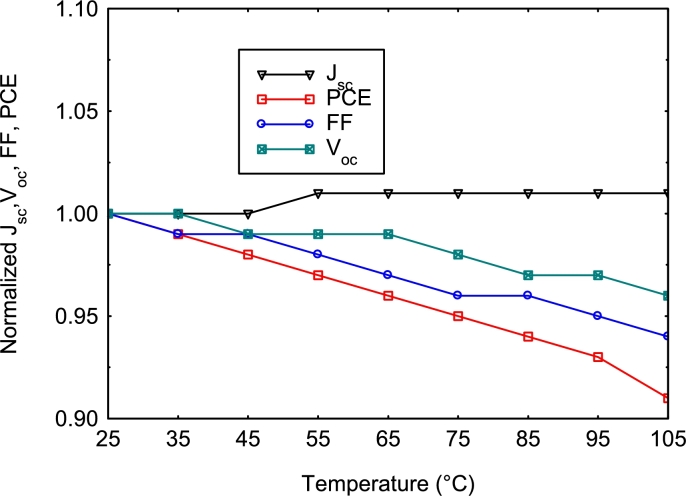
Normalized electrical parameters of the optimized solar cell as a function of operating temperature.

The normalized electrical parameters obtained are shown in Fig. 8. A slight increase in $J_{sc}$ is observed before reaching saturation. This increase is due to a decrease in the band gap with the temperature gradient (Equation (17)) and an increase in the number of charge carriers generated (Chander et al., 2015), and consequently, to a higher band-to-band absorption coefficient throughout the spectrum associated with the increased temperature (Sameera et al., 2022).

(17) $$E_{g}\left(T\right)=E_{g}\left(0\right)-\frac{\alpha T^{2}}{\left(T+\beta \right)}$$

The effect of temperature on $V_{oc}$ is due to the growth of the recombination process (Green, 2003; Jhuma et al., 2019). The *FF* and efficiency of the solar cell decrease with the increasing operating temperature due to the significant reduction of $V_{oc}$. Furthermore, based on the temperature coefficients of the efficiency curves, we established that the solar cell structure (Glass/SnO_2_:F/TiO_2_/MA_0.1_FA_0.9_Pb_0.85_Sr_0.15_I_3_/Cu_2_O/Au) obtained in this work is more stable than the Glass/SnO_2_:F/TiO_2_/MA_0.5_FA_0.5_PbI_3_/Cu_2_O/Au solar cell structure obtained by Abena et al. (2022), because the *TC* of −0.00107/°C calculated by Equation (16) in the cell with the MA_0.1_FA_0.9_Pb_0.85_Sr_0.15_I_3_ absorber is lower than that (−0.0014/°C) of the cell with the MA_0.5_FA_0.5_PbI_3_ absorber in absolute value.

## 4 Conclusions

In this study, to improve the stability and reduce the toxicity of the MAPbI_3_ absorber, we proceeded with a partial cation substitution of MA^+^ using FA^+^ and Pb^2+^ using Sr^2+^ to form the hybrid perovskite (MA_1−*x*_FA_*x*_Pb_1−*y*_Sr_*y*_I_3_). To realize this study by numerical simulation using the SCAPS-1D software, we first established and solved the correlation equation of the MA_1−*x*_FA_*x*_Pb_1−*y*_Sr_*y*_I_3_ absorber band gap with *x* and *y* proportions. Therefore, several configurations are possible for each band gap value. However, the choice of our absorber (MA_0.1_FA_0.9_Pb_0.85_Sr_0.15_I_3_, Eg = 1.60 eV) was based on several criteria such as: the optimal substitution of MA and Pb, the maximum electrical parameters, the maximum limitation of the band gap according to the Shockley-Queisser limit for simple n-p junction perovskite, and the difficulty in practice to realize the structures with 100% FA. Thus, the simulations realized on the different interest parameters of the structure Glass/SnO_2_:F/TiO_2_/MA_0.1_FA_0.9_Pb_0.85_Sr_0.15_I_3_/Cu_2_O/Au lead to the maximum efficiency of 29.0% (*Jsc* = 24.2 mA/cm^2^, *Voc* = 1.37 V, and FF=87.49%), which is a 2.33% gain compared to the initial structure with MA_0.5_FA_0.5_PbI_3_ as an absorber. Finally, the study of the effect of the operating temperature suggests that the device having MA_0.1_FA_0.9_Pb_0.85_Sr_0.15_I_3_ as absorber is slightly more stable (TC=−0.00107/°C) than that having MA_0.5_FA_0.5_PbI_3_ as absorber (TC=−0.0014/°C). In this study, although progress has been achieved in lead reduction, its substitution by more than 15% strontium cannot be performed due to the limitation of the experimental data available to develop the band gap equation model of the perovskite absorber.

## Declarations

### Author contribution statement

Aimé Magloire Ntouga Abena; A. Teyou Ngoupo, Dr.: Conceived and designed the experiments; Performed the experiments; Analyzed and interpreted the data; Contributed reagents, materials, analysis tools or data; Wrote the paper.

J.M.B. Ndjaka, Pr.: Analyzed and interpreted the data; Contributed reagents, materials, analysis tools or data.

### Funding statement

This research did not receive any specific grant from funding agencies in the public, commercial, or not-for-profit sectors.

### Data availability statement

Data included in article/supplementary material/referenced in article.

### Declaration of interests statement

The authors declare no conflict of interest.

### Additional information

No additional information is available for this paper.

## References

[br0010] Abega F.X.A., Ngoupo A.T., Ndjaka J.M.B. (2021). Numerical design of ultrathin hydrogenated amorphous silicon-based solar cell. Int. J. Photoenergy.

[br0020] Abena A.M.N., Ngoupo A.T., Abega F.X.A., Ndjaka J.M.B. (2022). Numerical investigation of solar cells based on hybrid organic cation perovskite with inorganic HTL via SCAPS-1D. Chin. J. Phys..

[br0030] Adjokatse S., Kahmann S., Duim H., Loi M.A. (2019). Effects of strontium doping on the morphological, structural, and photophysical properties of FASnI _3_ perovskite thin films. APL Mater..

[br0040] Ahmed S., Jannat F., Khan M.A.K., Alim M.A. (2021). Numerical development of eco-friendly Cs2TiBr6 based perovskite solar cell with all-inorganic charge transport materials via SCAPS-1D. Optik.

[br0050] An Y., Shang A., Cao G., Wu S., Ma D., Li X. (2018). Perovskite solar cells: optoelectronic simulation and optimization. Solar RRL.

[br0060] Burgelman M., Nollet P., Degrave S. (2000). Modelling polycrystalline semiconductor solar cells. Thin Solid Films.

[br0070] Chander S., Purohit A., Sharma A., Nehra S.P., Dhaka M.S. (2015). A study on photovoltaic parameters of mono-crystalline silicon solar cell with cell temperature. Energy Rep..

[br0080] Cho A.-N., Park N.-G. (2017). Impact of interfacial layers in perovskite solar cells. ChemSusChem.

[br0090] Chowdhury M.S., Shahahmadi S.A., Chelvanathan P., Tiong S.K., Amin N., Techato K., Nuthammachot N., Chowdhury T., Suklueng M. (2020). Effect of deep-level defect density of the absorber layer and n/i interface in perovskite solar cells by SCAPS-1D. Results Phys..

[br0100] Docampo P., Ball J.M., Darwich M., Eperon G.E., Snaith H.J. (2013). Efficient organometal trihalide perovskite planar-heterojunction solar cells on flexible polymer substrates. Nat. Commun..

[br0110] Eperon G.E., Leijtens T., Bush K.A., Prasanna R., Green T., Wang J.T.-W., McMeekin D.P., Volonakis G., Milot R.L., May R., Palmstrom A., Slotcavage D.J., Belisle R.A., Patel J.B., Parrott E.S., Sutton R.J., Ma W., Moghadam F., Conings B., Snaith H.J. (2016). Perovskite-perovskite tandem photovoltaics with optimized band gaps. Science.

[br0120] Gamal N., Sedky S.H., Shaker A., Fedawy M. (2021). Design of lead-free perovskite solar cell using Zn1-MgO as ETL: SCAPS device simulation. Optik.

[br0130] Geist J. (1979). Quantum efficiency of the pn junction in silicon as an absolute radiometric standard. Appl. Opt..

[br0140] Green M.A. (1982).

[br0150] Green M.A. (2003). General temperature dependence of solar cell performance and implications for device modelling. Prog. Photovolt..

[br0160] Gualdrón-Reyes A.F., Macias-Pinilla D.F., Masi S., Echeverría-Arrondo C., Agouram S., Muñoz-Sanjosé V., Rodríguez-Pereira J., Macak J.M., Mora-Seró I. (2021). Engineering Sr-doping for enabling long-term stable FAPb_1−x_Sr_x_I_3_ quantum dots with 100% photoluminescence quantum yield. J. Mater. Chem. C.

[br0170] Guirdjebaye N., Ouédraogo S., Ngoupo A.T., Tcheum G.M., Ndjaka J.M.B. (2019). Junction configurations and their impacts on Cu (In, Ga) Se2 based solar cells performances. Opto-Electron. Rev..

[br0180] He X., Guo P., Wu J., Tu Y., Lan Z., Lin J., Huang M. (2017). Hybrid perovskite by mixing formamidinium and methylammonium lead iodides for high-performance planar solar cells with efficiency of 19.41%. Sol. Energy.

[br0190] Hirasawa M., Ishihara T., Goto T., Uchida K., Miura N. (1994). Magnetoabsorption of the lowest exciton in perovskite-type compound (CH3NH3)PbI3. Physica B, Condens. Matter.

[br0200] Hoefler S.F. (2017). Progress on lead-free metal halide perovskites for photovoltaic applications: a review. Monatsh. Chem..

[br0210] Hossain M.I., Alharbi F.H., Tabet N. (2015). Copper oxide as inorganic hole transport material for lead halide perovskite based solar cells. Sol. Energy.

[br0220] Hu X., Zhang X., Liang L., Bao J., Li S., Yang W., Xie Y. (2014). High-performance flexible broadband photodetector based on organolead halide perovskite. Adv. Funct. Mater..

[br0230] Ito N., Kamarudin M.A., Hirotani D., Zhang Y., Shen Q., Ogomi Y., Iikubo S., Minemoto T., Yoshino K., Hayase S. (2018). Mixed sn–ge perovskite for enhanced perovskite solar cell performance in air. J. Phys. Chem. Lett..

[br0240] Jacobsson T.J., Pazoki M., Hagfeldt A., Edvinsson T. (2015). Goldschmidt's rules and strontium replacement in lead halogen perovskite solar cells: theory and preliminary experiments on CH_3_NH_3_SrI_3_. J. Phys. Chem. C.

[br0250] Jamal M.S., Shahahmadi S.A., Wadi M.A.A., Chelvanathan P., Asim N., Misran H., Hossain M.I., Amin N., Sopian K., Akhtaruzzaman M. (2019). Effect of defect density and energy level mismatch on the performance of perovskite solar cells by numerical simulation. Optik.

[br0260] Jeyakumar R., Bag A., Nekovei R., Radhakrishnan R. (2020). Influence of electron transport layer (TiO2) thickness and its doping density on the performance of CH3NH3PbI3-based planar perovskite solar cells. J. Electron. Mater..

[br0270] Jhuma F.A., Shaily M.Z., Rashid M.J. (2019). Towards high-efficiency CZTS solar cell through buffer layer optimization. Mater. Renew. Sustain. Energy.

[br0280] Kapil G., Bessho T., Ng C.H., Hamada K., Pandey M., Kamarudin M.A., Hirotani D., Kinoshita T., Minemoto T., Shen Q., Toyoda T., Murakami T.N., Segawa H., Hayase S. (2019). Strain relaxation and light management in tin–lead perovskite solar cells to achieve high efficiencies. ACS Energy Lett..

[br0690] Kour R., Arya S., Verma S., Gupta J., Bandhoria P., Bharti V., Datt R., Gupta V. (2019). Potential substitutes for replacement of lead in perovskite solar cells: a review. Glob. Chall..

[br0290] Krajangsang T., Moollakorn A., Inthisang S., Limmanee A., Sriprapha K., Boriraksantikul N., Taratiwat T., Akarapanjavit N., Sritharathikhun J. (2014). Study of an amorphous silicon oxide buffer layer for p-type microcrystalline silicon oxide/n-type crystalline silicon heterojunction solar cells and their temperature dependence. Int. J. Photoenergy.

[br0300] Lau C.F.J., Zhang M., Deng X., Zheng J., Bing J., Ma Q., Kim J., Hu L., Green M.A., Huang S. (2017). Strontium-doped low-temperature-processed CsPbI2Br perovskite solar cells. ACS Energy Lett..

[br0310] Lin L., Jiang L., Li P., Fan B., Qiu Y., Yan F. (2019). Simulation of optimum band structure of HTM-free perovskite solar cells based on ZnO electron transporting layer. Mater. Sci. Semicond. Process..

[br0320] Lin R., Xiao K., Qin Z., Han Q., Zhang C., Wei M., Saidaminov M.I., Gao Y., Xu J., Xiao M., Li A., Zhu J., Sargent E.H., Tan H. (2019). Monolithic all-perovskite tandem solar cells with 24.8% efficiency exploiting comproportionation to suppress Sn(ii) oxidation in precursor ink. Nat. Energy.

[br0330] MaríSoucase B., Pradas I.G., Adhikari K.R. (2016). Perovskite Materials: Synthesis, Characterisation, Properties, and Applications.

[br0340] Mateen M., Arain Z., Liu X., Liu C., Yang Y., Ding Y., Ma S., Ren Y., Wu Y., Tao Y. (2020). High-performance mixed-cation mixed-halide perovskite solar cells enabled by a facile intermediate engineering technique. J. Power Sources.

[br0350] McMeekin D.P., Sadoughi G., Rehman W., Eperon G.E., Saliba M., Hörantner M.T., Haghighirad A., Sakai N., Korte L., Rech B., Johnston M.B., Herz L.M., Snaith H.J. (2016). A mixed-cation lead mixed-halide perovskite absorber for tandem solar cells. Science.

[br0360] Mozaffari M., Behjat A., Mirjalili B.F. (2018). The effect of solution process control on the formation of the *α*-FAPbI3 perovskite: FAPbI3 versus MAPbI3 solar cells. Sol. Energy.

[br0370] Navas J., Sánchez-Coronilla A., Gallardo J.J., Hernández N.C., Piñero J.C., Alcántara R., Fernández-Lorenzo C., Desireé M., Aguilar T., Martín-Calleja J. (2015). New insights into organic–inorganic hybrid perovskite CH3NH3PbI3 nanoparticles. An experimental and theoretical study of doping in Pb2+ sites with Sn2+, Sr2+, Cd2+ and Ca2+. Nanoscale.

[br0380] Ngoupo A.T., Ouédraogo S., Zougmoré F., Ndjaka J.M.B. (2021). Numerical analysis of ultrathin Sb2Se3-based solar cells by SCAPS-1D numerical simulator device. Chin. J. Phys..

[br0390] Noel N.K., Stranks S.D., Abate A., Wehrenfennig C., Guarnera S., Haghighirad A.-A., Sadhanala A., Eperon G.E., Pathak S.K., Johnston M.B. (2014). Lead-free organic–inorganic tin halide perovskites for photovoltaic applications. Energy Environ. Sci..

[br0400] Ono L.K., Juarez-Perez E.J., Qi Y. (2017). Progress on perovskite materials and solar cells with mixed cations and halide anions. ACS Appl. Mater. Interfaces.

[br0410] Pellet N., Gao P., Gregori G., Yang T.-Y., Nazeeruddin M.K., Maier J., Grätzel M. (2014). Mixed-organic-cation perovskite photovoltaics for enhanced solar-light harvesting. Angew. Chem..

[br0420] Pérez-del-Rey D., Forgács D., Hutter E.M., Savenije T.J., Nordlund D., Schulz P., Berry J.J., Sessolo M., Bolink H.J. (2016). Strontium insertion in methylammonium lead iodide: long charge carrier lifetime and high fill-factor solar cells. Adv. Mater..

[br0430] Polman A., Knight M., Garnett E.C., Ehrler B., Sinke W.C. (2016). Photovoltaic materials: present efficiencies and future challenges. Science.

[br0440] Rai S., Pandey B.K., Dwivedi D.K. (2020). Modeling of highly efficient and low cost CH3NH3Pb (I1-xClx) 3 based perovskite solar cell by numerical simulation. Opt. Mater..

[br0450] Saliba M., Matsui T., Seo J.-Y., Domanski K., Correa-Baena J.-P., Nazeeruddin M.K., Zakeeruddin S.M., Tress W., Abate A., Hagfeldt A., Grätzel M. (2016). Cesium-containing triple cation perovskite solar cells: improved stability, reproducibility and high efficiency. Energy Environ. Sci..

[br0460] Sameera J.N., Islam M.A., Islam S., Hossain T., Sobayel M.K., Akhtaruzzaman M., Amin N., Rashid M.J. (2022). Cubic silicon carbide (3C–SiC) as a buffer layer for high efficiency and highly stable CdTe solar cell. Opt. Mater..

[br0470] Sawicka-Chudy P., Sibiński M., Wisz G., Rybak E., Cholewa M. (2017). Numerical analysis and optimization of Cu2O/TiO2, CuO/TiO2, heterojunction solar cells using SCAPS. J. Phys..

[br0480] Scharber M.C., Mühlbacher D., Koppe M., Denk P., Waldauf C., Heeger A.J., Brabec C.J. (2006). Design rules for donors in bulk-heterojunction solar cells—towards 10% energy-conversion efficiency. Adv. Mater..

[br0490] Sengar B.S., Garg V., Kumar A., Dwivedi P. (2021). Numerical simulation: design of high-efficiency planar pn homojunction perovskite solar cells. IEEE Trans. Electron Devices.

[br0500] Shockley W., Queisser H.J. (1961). Detailed balance limit of efficiency of p-n junction solar cells. J. Appl. Phys..

[br0510] Si F., Tang F., Xue H., Qi R. (2016). Effects of defect states on the performance of perovskite solar cells. J. Semicond..

[br0520] Slami A., Bouchaour M., Merad L. (2019). Numerical study of based perovskite solar cells by SCAPS-1D. Int. J. Energy Environ..

[br0530] Sridharan A., Noel N.K., Hwang H., Hafezian S., Rand B.P., Kéna-Cohen S. (2019). Time-resolved imaging of non-diffusive carrier transport in long-lifetime halide perovskite thin films. arxiv:1905.11242.

[br0540] Swafford L.A., Weigand L.A., Bowers M.J., McBride J.R., Rapaport J.L., Watt T.L., Dixit S.K., Feldman L.C., Rosenthal S.J. (2006). Homogeneously alloyed CdS x Se1-x nanocrystals: synthesis, characterization, and composition/size-dependent band gap. J. Am. Chem. Soc..

[br0550] Tan K., Lin P., Wang G., Liu Y., Xu Z., Lin Y. (2016). Controllable design of solid-state perovskite solar cells by SCAPS device simulation. Solid-State Electron..

[br0560] Tong J., Song Z., Kim D.H., Chen X., Chen C., Palmstrom A.F., Ndione P.F., Reese M.O., Dunfield S.P., Reid O.G., Liu J., Zhang F., Harvey S.P., Li Z., Christensen S.T., Teeter G., Zhao D., Al-Jassim M.M., van Hest M.F.A.M., Zhu K. (2019). Carrier lifetimes of >1 μs in Sn-Pb perovskites enable efficient all-perovskite tandem solar cells. Science.

[br0570] Travis W., Glover E.N.K., Bronstein H., Scanlon D.O., Palgrave R.G. (2016). On the application of the tolerance factor to inorganic and hybrid halide perovskites: a revised system. Chem. Sci..

[br0580] Vandewal K., Gadisa A., Oosterbaan W.D., Bertho S., Banishoeib F., Van Severen I., Lutsen L., Cleij T.J., Vanderzande D., Manca J.V. (2008). The relation between open-circuit voltage and the onset of photocurrent generation by charge-transfer absorption in polymer: fullerene bulk heterojunction solar cells: photocurrent generation by charge-transfer absorption. Adv. Funct. Mater..

[br0590] Venugopal R., Lin P.-I., Chen Y.-T. (2006). Photoluminescence and Raman scattering from catalytically grown Zn_*x*_Cd${}_{1-x}$Se alloy nanowires. J. Phys. Chem. B.

[br0600] Wang M., Fei G.T., Zhang Y.G., Kong M.G., Zhang L.D. (2007). Tunable and predetermined bandgap emissions in alloyed ZnSxSe1–x nanowires. Adv. Mater..

[br0610] Xu J., Yang X., Wang H., Chen X., Luan C., Xu Z., Lu Z., Roy V.A.L., Zhang W., Lee C.-S. (2011). Arrays of ZnO/Zn_*x*_Cd_1−*x*_Se nanocables: band gap engineering and photovoltaic applications. Nano Lett..

[br0620] Xu X., Chueh C.-C., Yang Z., Rajagopal A., Xu J., Jo S.B., Jen A.K.-Y. (2017). Ascorbic acid as an effective antioxidant additive to enhance the efficiency and stability of Pb/Sn-based binary perovskite solar cells. Nano Energy.

[br0630] Yang Z., Rajagopal A., Jen A.K.-Y. (2017). Ideal bandgap organic–inorganic hybrid perovskite solar cells. Adv. Mater..

[br0640] Yang Z., Yu Z., Wei H., Xiao X., Ni Z., Chen B., Deng Y., Habisreutinger S.N., Chen X., Wang K., Zhao J., Rudd P.N., Berry J.J., Beard M.C., Huang J. (2019). Enhancing electron diffusion length in narrow-bandgap perovskites for efficient monolithic perovskite tandem solar cells. Nat. Commun..

[br0650] Yin W.-J., Shi T., Yan Y. (2014). Unusual defect physics in CH_3_NH_3_PbI _3_ perovskite solar cell absorber. Appl. Phys. Lett..

[br0660] Zhou Y., Chen J., Bakr O.M., Sun H.-T. (2018). Metal-doped lead halide perovskites: synthesis, properties, and optoelectronic applications. Chem. Mater..

[br0670] Zhu L., Shao G., Luo J.K. (2011). Numerical study of metal oxide heterojunction solar cells. Semicond. Sci. Technol..

[br0680] Zhu Z., Li N., Zhao D., Wang L., Jen A.K.-Y. (2019). Improved efficiency and stability of Pb/Sn binary perovskite solar cells fabricated by galvanic displacement reaction. Adv. Energy Mater..

